# Extraction with Gas

**Published:** 1883-06

**Authors:** 


Extraction with Gas.—"There is nothing very brilliant about our Bremen dentists," recently said a young lady to a member of the profession at Berlin, "but they are very obliging. If you wish a tooth extracted with gas, they forthwith light the chandelier."—*Zahntechnische Reform.*

